# Response of *Zostera japonica* rhizosphere bacteria to ocean acidification

**DOI:** 10.1128/aem.00277-26

**Published:** 2026-06-29

**Authors:** Xinqi Li, Yu Zang, Hongzhen Wang, Jiayi Xin, Lei Liu, Yuhui Zhang, Wenqin Chen, Xiying Meng, Qifan Zhang, Xuexi Tang, Jun Chen

**Affiliations:** 1 MoE Key Laboratory of Evolution & Marine Biodiversity, College of Marine Life Sciences, Ocean University of China, Qingdao, Shandong, China; 2 Key Laboratory of Marine Eco-Environmental Science and Technology, First Institute of Oceanography, Ministry of Natural Resources, Qingdao, Shandong, China; The University of Arizona, Tucson, Arizona, USA

**Keywords:** *Zostera japonica*, rhizosphere, bacterial community, bacterial function prediction

## Abstract

Amidst global climate change, the escalating atmospheric CO_2_ levels have intensified ocean acidification (OA), significantly impacting the structure and function of marine ecosystems. Seagrass beds, representative nearshore ecosystems, play a pivotal role in carbon sequestration, biodiversity preservation, and nearshore environmental equilibrium. Rhizosphere microorganisms within seagrass beds, essential components of the ecosystem, drive material cycling and energy flow. Their community structure and functions demonstrate heightened sensitivity to environmental variations. While previous studies have primarily focused on the effects of ocean acidification on seagrass hosts, limited attention has been given to the rhizosphere. Therefore, this study selected *Zostera japonica* as the focal species and systematically evaluated changes in the structure and function of the rhizosphere bacterial community across varying acidification levels (400 ppm, 1,000 ppm, 2,000 ppm CO_2_) within an ocean acidification context. The results revealed a significant decline in the richness and diversity of the rhizosphere bacterial community under acidification, accompanied by shifts in community composition characterized by an increase in the relative abundance of Bacteroidota and *Tenacibaculum* with escalating acidification levels. In high acidification conditions, bacterial network interactions exhibited a trend toward simplification; yet the number of key taxonomic units increases, and there was a shift in community assembly from stochastic to deterministic processes. Functional predictions indicated the enhancement of microbial carbon sequestration and nitrogen fixation under acidification, while denitrification and specific sulfur metabolism pathways were inhibited. This implies that in acidified environments, the rhizosphere bacterial community may enhance carbon and nitrogen fixation to uphold nutrient supply.

**IMPORTANCE:**

Against the background of escalating global climate change and ocean acidification, seagrass beds, as crucial blue carbon sink ecosystems, face formidable challenges to their ecological functions and stability. Rhizosphere microorganisms of seagrasses, serving as the “second genome” of the seagrass host, play a central role in material cycling, nutrient supply, and system stability within seagrass beds. They are a key biological component that supports seagrass adaptation to environmental changes. Therefore, investigating the response and adaptation mechanisms of seagrass rhizosphere bacterial communities under ocean acidification is essential for deepening our understanding of the stability and resilience of seagrass bed ecosystems.

## INTRODUCTION

Various parts of plants (such as roots, stems, leaves, flowers, and fruits) harbor highly diverse microbial communities (referred to as plant microbiome), and the interactions between plants and their microbiomes are crucial for the host’s ability to adapt to the environment (1–5). The relationship between plants and their microbiomes is not static but rather influenced by the environment to some extent. Studies have shown that environmental factors can significantly impact the community structure and function of plant microbiomes (6–9). Currently, the Earth’s ecosystems are undergoing rapid climate change characterized by rising atmospheric CO_2_ levels, global temperature increases, and frequent extreme precipitation events. These challenges have profound effects on plants, microbes, their interactions, and ecosystem processes and functions (10–13).

Among the various factors of global climate change, ocean acidification driven by the continuous increase in atmospheric CO_2_ concentration is considered one of the major threats to marine resources (14). Studies have shown that since the Industrial Revolution, surface seawater pH has decreased by approximately 0.1 units, corresponding to an increase in atmospheric CO_2_ partial pressure (pCO_2_) from around 280 ppm to the current level of about 400 ppm (15). Under the “business as usual” scenario (RCP 8.5), surface seawater pCO_2_ is projected to rise further to approximately 1,000 ppm by 2100 (16), with pH potentially declining by an additional 0.3 units and pCO_₂_ potentially reaching 2,000 ppm by 2300 (17). This rapid change in the chemical environment poses significant threats to the diversity of habitat-building organisms and calcifying organisms (18, 19). However, recent studies have found that non-calcifying marine autotrophs such as seagrasses are expected to benefit from increased CO_2_ and bicarbonate ion supply, promoting their photosynthesis (20–24). Moreover, as key drivers of marine ecosystem functioning, marine microorganisms exhibit pronounced responses to ocean acidification. Previous studies have demonstrated that acidification can significantly alter the composition of planktonic bacterial communities, with its effects being more persistent than those of warming alone (25). In mesocosm experiments conducted in eutrophic subtropical waters, elevated CO_2_ conditions (1,000 ppm) led to marked changes in the complexity and stability of co-occurrence networks in planktonic bacterial communities, while epiphytic bacteria were found to be more sensitive to high CO_2_ levels than their planktonic counterparts (26).

Seagrasses play essential ecological roles in marine environments, performing many vital functions in ecosystems (27, 28). Similar to terrestrial plants, seagrasses (including their roots, stems, leaf surfaces, and rhizospheric sediments) harbor complex and specific microbial communities that play crucial roles in nutrient absorption, disease defense, carbon and nitrogen cycling, and responding to environmental impacts (1, 29, 30). The rhizosphere is a biologically active region with different physicochemical properties from the surrounding soil (31). Plants provide suitable microhabitats for various microorganisms through root exudates (32). Additionally, the rhizosphere microbial genome is considered the plant’s “second genome,” reflecting the plant’s ecological adaptation strategies (33) and ecological functional potential (34). Zhang et al. (35) demonstrated that, under the combined stress of ocean warming and acidification, the stability of bacterial communities associated with the seagrass *Thalassia hemprichii* increased, accompanied by a significant enrichment of genes involved in sulfur metabolism (35). However, previous studies have predominantly focused on the direct effects of acidification on seagrass hosts, whereas the rhizosphere microbiome, one of the most sensitive components of seagrass ecosystems to environmental change, has received comparatively limited attention. Therefore, this study selected *Zostera japonica*, one of the key supporting species in seagrass beds, as the research object. By constructing three representative acidification gradient models of 400 ppm, 1,000 ppm, and 2,000 ppm CO_2_, this study systematically investigated the effects of ocean acidification on the rhizosphere microbes of *Zostera japonica* from the aspects of community structure, interaction networks, assembly mechanisms, and functional predictions. By integrating physiological and ecological indicators of seagrass hosts, the study aims to elucidate the role of bacterial community responses in seagrass adaptation to acidification stress, providing a microbial perspective theoretical basis for predicting and assessing the fate of seagrass ecosystems under future climate change.

## MATERIALS AND METHODS

### Experimental design

*Zostera japonica* plants were randomly collected from the intertidal seagrass meadows in Huiquan Bay, Qingdao (36.05,065 N, 120.3439 E). Subsequently, the samples were placed in shaded containers filled with seawater and transported to the laboratory for acclimatization for 72 h before acidification treatment. The acidification system was based on a 5,000-liter closed mixing tank, where CO_2_ injection was precisely controlled using a non-dispersive infrared gas analyzer (IRGA) and a PID digital controller to maintain three pCO_2_ levels: 400 ppm, 1,000 ppm, and 2,000 ppm, corresponding to the C, M, and H groups, respectively. The indoor cultivation experiment was conducted in a temperature-controlled greenhouse from 28 August 28 2022 to 6 October 2022 (Fig. S1A), totaling 40 days. The detailed description of the acidification system design has been previously published in our work (36).

### Sample collection, environmental physicochemical parameters, and seagrass physiological measurements

After 40 days, the leaf length and maximum quantum yield (Fv/Fm) of each *Zostera japonica* sample were measured. The maximum quantum yield (Fv/Fm) was assessed using a chlorophyll fluorescence imaging system (MINI-PAM-II; Heinz Walz, Effeltrich, Germany). Approximately 10 individuals of *Zostera japonica* were planted in each culture tank across all treatment groups. From each treatment, eight individuals were randomly selected for rhizosphere bacterial sampling (Fig. S1B), yielding a total of 24 samples (8 replicates × 3 treatments).

For rhizosphere sample collection, a common method used for collecting microbial samples from terrestrial plant rhizospheres was employed (37–39). Selected seagrass roots were manually shaken to remove loose sediments, and the remaining sediments attached to the rhizosphere were collected with a needle after rinsing with seawater. All samples were collected in cryovials, immediately stored in liquid nitrogen on-site, and then transferred to a −80°C freezer in the laboratory for storage until DNA extraction. The determination of environmental factors and seagrass physiological parameters in each treatment group followed the specific indicators and methods described in our previous publication (36).

### High-throughput sequencing and raw data processing

Total DNA was extracted from each sample using the FastDNA SPIN Kit (MP Biomedicals, Solon, United States). The quality and concentration of the DNA extracts were assessed using a NanoDrop 2000, and the integrity of the DNA was confirmed by agarose gel electrophoresis.

Bacterial primers 338F (5′-ACTCCTACGGGAGGCAGCAG-3′) and 806R (5′-GGACTACHVGGGTWTCTAAT-3′) were used to amplify the V3–V4 region of the 16S rDNA. PCR amplification was performed on an ABI GeneAmp 9700 PCR machine (Perkin-Elmer, United States) with the following program: 3 min at 95°C for initial denaturation; 30 s at 95°C for denaturation, 30 s at 55°C for annealing, and 45 s at 72°C for extension, for a total of 30 cycles; and a final extension at 72°C for 10 min. Purified PCR products were then subjected to library construction using the NEXTFLEX Rapid DNA-Seq Kit. Sequencing was performed on the Illumina Nextseq2000 platform. Raw paired-end sequences were quality-filtered using fastp (40) (https://github.com/OpenGene/fastp, version 0.19.6) and subsequently merged with FLASH (41) (http://www.cbcb.umd.edu/software/flash, version 1.2.11). The resulting high-quality sequences were merged using the DADA2 plugin within the QIIME2 pipeline (42) with default parameters for denoising. Sequences with an abundance of fewer than five were removed (43), yielding the final amplicon sequence variants (ASVs) (Table S1). Chloroplast and mitochondrial sequences were excluded from all samples prior to downstream analyses. Before calculating bacterial diversity, the ASV table was rarefied to the minimum sequencing depth across samples to minimize biases associated with uneven sequencing effort (44).

After rarefaction, the average sequence coverage (Good’s coverage) across all samples remained at 99.09%. Taxonomic classification of ASVs was performed using the Sliva 16S rRNA gene database (v 138) with the Blast classifier within Qiime2. Functional predictions of 16S data were analyzed using PICRUSt2 (45, 46).

### Statistical analysis

Alpha diversity indices, such as Sobs, Simpson, and Pielou indices, were calculated using mothur software (http://www.mothur.org/wiki/Calculators), and Wilcoxon rank-sum tests were performed for intergroup differences in alpha diversity (42). Differences in community composition among the three treatment groups were assessed using one-way analysis of variance (ANOVA). Multiple testing was controlled using the false discovery rate (FDR), followed by Tukey-Kramer post hoc tests for pairwise comparisons, with a confidence level set at 0.95. NMDS analysis based on the Bray-Curtis distance algorithm was used to assess the similarity of bacterial community structures among samples, and PERMANOVA non-parametric tests were employed to analyze the significance of differences in bacterial community structures among sample groups. Co-occurrence network analysis was performed using Spearman’s correlations, calculated among the top 99% ASVs based on relative abundance at the ASV classification level. The analysis was performed using the psych package in R (47) with significance set at *P*  <  0.05 and a correlation coefficient threshold of |R|  >  0.7 (48, 49). The results were visually represented using Gephi (50). Null models were used to evaluate the relative contributions of deterministic and stochastic processes in bacterial community assembly (51, 52). Systematic phylogenetic turnover and null models were compared to analyze significant phylogenetic signals in bacterial community assembly mechanisms (53). The phylogenetic distance between different ASVs was calculated using the picante package, and the strength of the phylogenetic signal was evaluated by comparing the phylogenetic distance matrix with the differential matrix of optimal environmental parameters. The Spearman correlation coefficient of phylogenetic distances of rhizosphere bacterial communities of *Zostera japonica* under acidification conditions was calculated using the “mantel.correlog” function in the vegan package (54). Through one-way ANOVA, differences in genes related to carbon fixation, nitrogen metabolism, and sulfur metabolism were identified in rhizosphere samples based on functional predictions. Multiple testing was performed using FDR, with Tukey-Kramer post-hoc tests applied, and the confidence level set at 0.95.

## RESULTS

### Environmental factors and response of *Zostera japonica* to ocean acidification

The results indicated that, with increasing levels of acidification, the concentrations of dissolved inorganic carbon (DIC) and bicarbonate (HCO_3_⁻) in the seawater significantly increased (Fig. S1C). The leaf length of *Zostera japonica* showed a significant increase, while ocean acidification led to a significant decrease in the maximum photosynthetic efficiency of Photosystem II (Fv/Fm).

### Bacterial community diversity and composition

**Fig 1:**
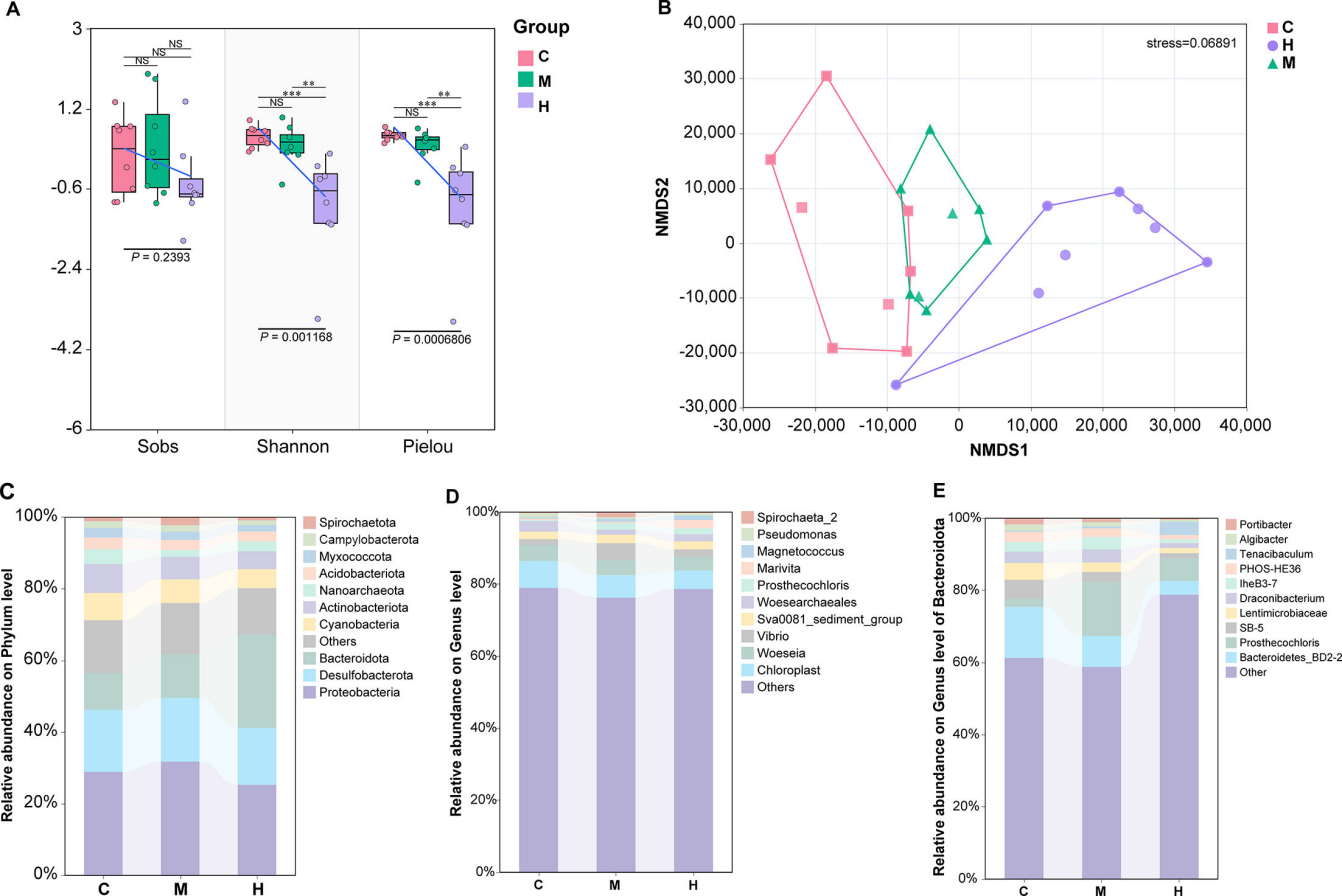
Under three acidification conditions, the rhizosphere bacterial community structure of *Zostera japonica* was analyzed (C indicates the 400 ppm CO_2_ treatment group; M indicates the 1,000 ppm CO_2_ treatment group; H indicates the 2,000 ppm CO_2_ treatment group). (**A**) Results of alpha diversity indices of the rhizosphere bacterial community. (**B**) Non-metric multidimensional scaling (NMDS) of the bacterial community structure based on the Bray-Curtis distance matrix. (**C**) Relative abundance of the bacterial community at the phylum level. (**D**) Relative abundance of the bacterial community at the genus level. (**E**) Relative abundance of the bacterial community at the genus level within the Bacteroidota phylum. Boxplot showing 24 samples (*n* = 24). Statistical comparisons of the data were conducted using the Wilcoxon rank-sum test. Significance levels are denoted as follows: *P < 0.05, **P < 0.01, ***P < 0.001.

A total of 1,497,172 high-quality sequences were obtained from bacterial community analysis. In total, there were 16,838 amplicon sequence variants (ASVs) across all samples. The results revealed a significant decrease in the richness (Wilcoxon rank-sum test, *P* < 0.05) and diversity (Wilcoxon rank-sum test, *P* < 0.05) of the rhizosphere bacterial community of *Zostera japonica* under different acidification treatments (Fig. 1A). Notably, under the 1,000 ppm treatment, the richness and diversity of the rhizosphere bacterial community showed no significant difference compared to the C group. NMDS analysis demonstrated distinct differences in the bacterial community composition of rhizosphere samples under different acidification treatments (Fig. 1B). Additionally, PERMANOVA results confirmed the significant impact of acidification on the composition of seagrass bacterial communities (Table S2).

The community composition analysis revealed that the dominant phylum in the rhizosphere bacterial community was Proteobacteria, Desulfobacterota, and Bacteroidota (Fig. 1C). At the genus level, the predominant genera were *Chloroplast*, *Woeseia*, and *Vibrio* (Fig. 1D). Notably, we observed that the relative abundance of Bacteroidota increased significantly with increasing levels of acidification (one-way ANOVA, *P* < 0.05). Based on this, we examined the relative abundance of the bacterial community at the genus level within the Bacteroidota phylum and found that only the genus *Tenacibaculum* exhibited a significant increase in relative abundance along the acidification gradient (one-way ANOVA, *P* < 0.05).

### Interactions within the bacterial community and community assembly process

**Fig 2:**
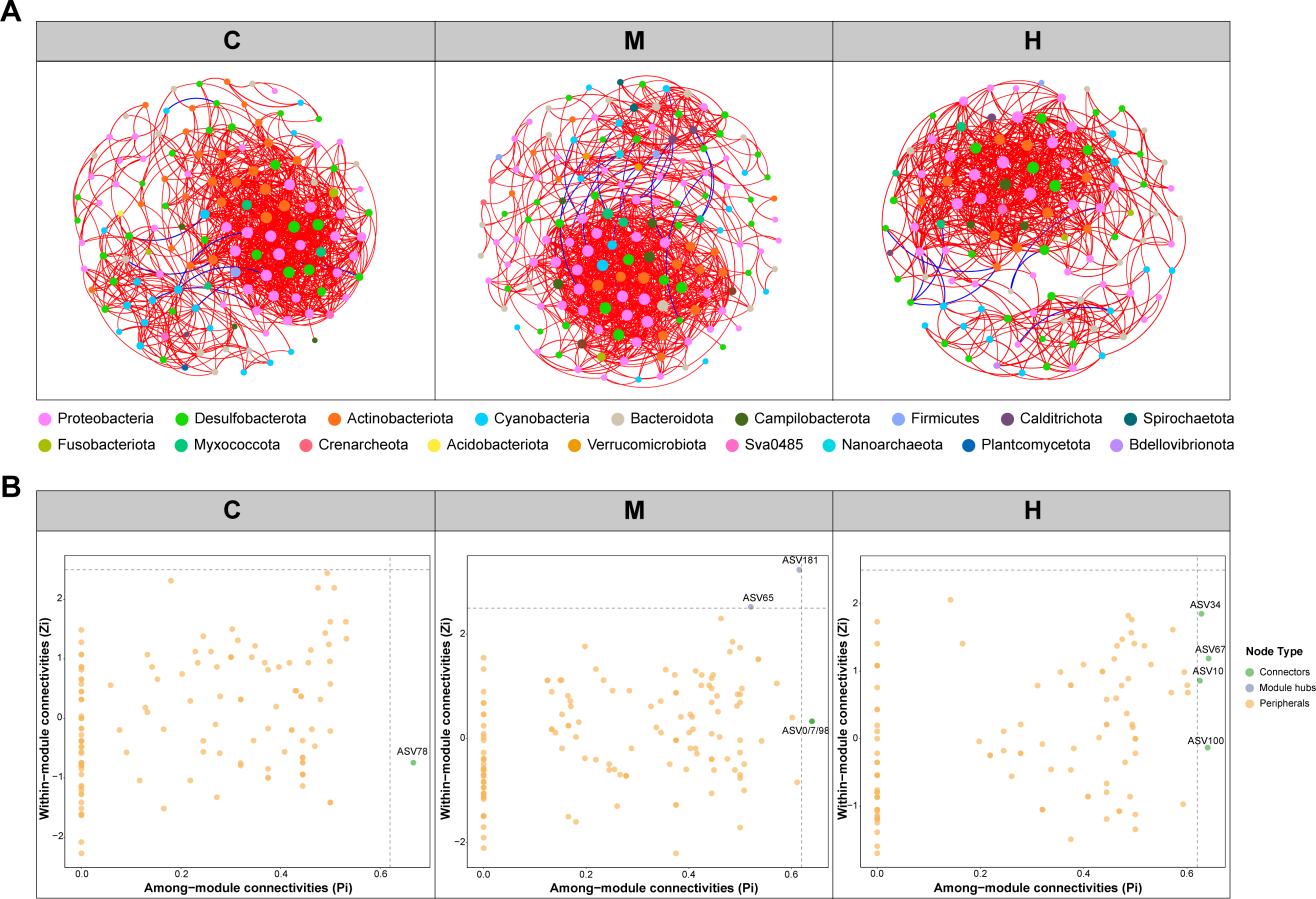
Co-occurrence network (**A**) and key taxa (**B**) of bacterial communities at the ASV levels under different acidification treatments. (**A**) The top 99% ASVs for relative abundance were selected for each sample. Nodes in the network are color-coded for different bacterial phyla, while lines connecting nodes represent correlations between ASVs. Positive correlations are indicated by red lines and negative ones by blue lines. (**B**) Module hubs were defined as taxa with *Zi* ≥ 2.5 and *Pi* < 0.62, connectors as taxa with *Zi* < 2.5 and *Pi* ≥ 0.62, and network hubs as taxa with *Zi* ≥ 2.5 and *Pi* ≥ 0.62.

In addition, we constructed co-occurrence networks of rhizosphere bacterial communities based on the top 99% most abundant ASVs (Fig. 2A). Compared with the C and M groups, the H group exhibited lower numbers of nodes, edges, and reduced modularity (Table S3). This pattern suggests that high-level acidification led to substantial declines in the abundance of certain taxa, thereby reducing the number of network nodes as environmental stress increased. Moreover, under high acidification, the interactions among rhizosphere bacteria were diminished, resulting in a simplified network structure. This observation is consistent with the reduced modularity observed under the 2,000 ppm treatment, highlighting the impact of environmental stress on biodiversity and biotic interactions. Subsequently, based on topological roles, we further examined shifts in keystone taxa within the seagrass rhizosphere bacterial community (Fig. 2B). Compared with the 400 ppm treatment, the number of keystone taxa increased markedly in both the 1,000 ppm and 2,000 ppm treatments, with most of these taxa being associated with carbon and nitrogen cycling (Table S4).

**Fig 3:**
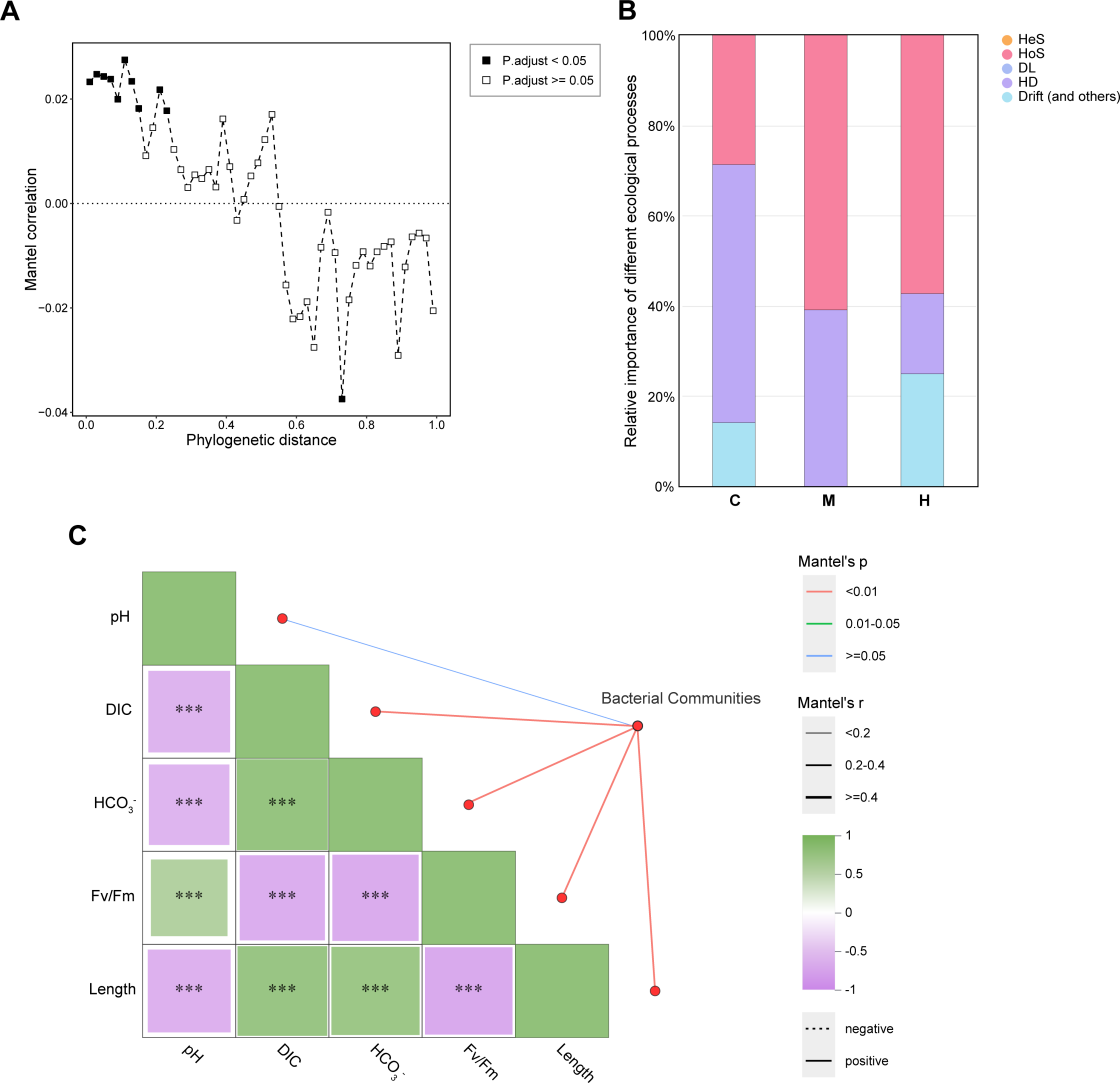
The assembly mechanism of the rhizosphere bacterial community and its correlation with environmental factors were analyzed under different acidification conditions. (**A**) Mantel correlograms show significant phylogenetic signals at short phylogenetic distances. Black squares indicate significant spatial autocorrelation relationships (*P* < 0.05) within that distance range. (**B**) Community assembly processes of the bacterial community from different groups. (**C**) The Spearman correlation among different environmental variables and the correlation between environmental variables and the bacterial community structure based on the Mantel test were assessed (DIC, dissolved inorganic carbon; Fv/Fm, maximum photosynthetic efficiency of Photosystem II; Length, length of *Zostera japonica* leaves).

In the analysis of bacterial community assembly mechanisms (using models such as βMNTD and βNTI), a critical premise is to validate the presence of a significant phylogenetic signal. This signal indicates that species phylogenetic relationships are conservative with their ecological characteristics (such as environmental preferences), meaning closely related species have more similar ecological traits. The calculation of this signal aims to assess whether the observed phylogenetic turnover patterns can be effectively used for ecological inference; only when a significant phylogenetic signal is present, the results of comparing phylogenetic distance-based null models have reliable ecological significance, enabling the accurate resolution of the relative roles of deterministic and stochastic processes in community assembly. Strong phylogenetic signal, as indicated by statistical tests, implies closer relationships among taxonomic units with close phylogenetic proximity, indicating phylogenetic clustering, while a weak signal suggests less close relationships among taxonomic units, indicating phylogenetic over-dispersion or evenness, possibly caused by processes such as convergent evolution. In this study, we calculated the phylogenetic distance between different ASVs, evaluated the strength of the phylogenetic signal by comparing the phylogenetic distance matrix with the differential matrix of optimal environmental parameters, and calculated the Spearman correlation coefficient of phylogenetic distances of rhizosphere bacterial communities of *Zostera japonica* under acidification conditions. All Mantel correlograms exhibited significant positive correlations at short phylogenetic distances (Fig. 3A), indicating that, under acidification, co-occurring rhizosphere ASVs tend to be phylogenetically clustered. This pattern suggests that closely related ASVs share similar ecological preferences within the rhizosphere environment. By using null models, we investigated the community assembly process of rhizosphere bacterial communities of *Zostera japonica* under different acidification conditions. The results revealed that in the rhizosphere, as the level of acidification increased, from 1,000 ppm onward, deterministic processes (|βNTI| > 2) dominated the construction of the bacterial community (Fig. 3B).

### Environmental factors and functional predictions impacting microbial communities

Subsequently, we tested the influence of different environmental and biological factors on the rhizosphere bacterial community (Fig. 3C). Specifically, various measured environmental and biological factors significantly affected the bacterial community in the rhizosphere samples (except pH). Additionally, the interplay between environmental factors revealed significant negative correlations of DIC, HCO_3_⁻, and leaf elongation with pH, while only the maximum photosynthetic efficiency of Photosystem II (Fv/Fm) showed a significant positive correlation with pH. This indicates that, as acidification levels increase, DIC, HCO_3_⁻, and leaf elongation increase, while the maximum photosynthetic efficiency of *Zostera japonica* (Fv/Fm) decreases. This suggests that ocean acidification significantly enhances the availability of inorganic carbon in the rhizosphere environment and affects the growth and photosynthetic performance of *Zostera japonica*.

**Fig 4:**
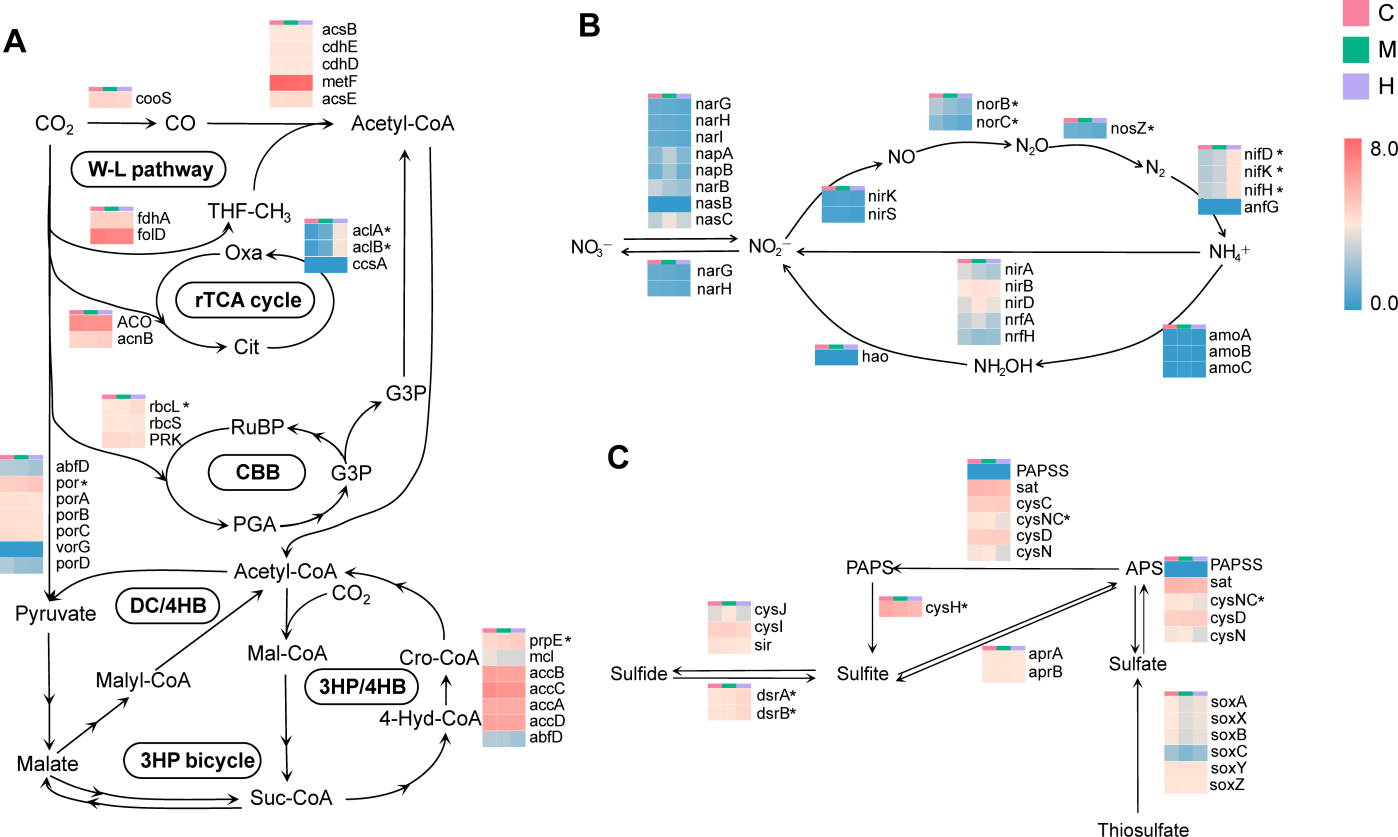
Metabolic activities of bacterial communities in the rhizosphere of *Zostera japonica* based on functional prediction analysis. (**A**) Schematic diagram of the carbon fixation pathway. (**B**) Schematic diagram of the nitrogen metabolism pathway. (**C**) Schematic diagram of the sulfur metabolism pathway. The heatmap displays the enrichment of functional genes involved in carbon fixation, nitrogen metabolism, and sulfur metabolism by rhizosphere microbes under different acidification treatments. Significance of gene abundance changes (*P* < 0.05) is indicated by asterisks (*).

To further investigate how environmental changes drive the functional responses of bacterial communities, we used PICRUSt2 to predict the functional potential of the bacterial community and focused on analyzing metabolic pathways related to key biogeochemical cycles. It is important to note that the results presented in this study are based on predicted gene abundances from 16S rRNA gene sequences, rather than directly measured gene abundances. The findings reveal that ocean acidification significantly reshapes the metabolic functional characteristics of rhizospheric bacteria related to carbon, nitrogen, and sulfur metabolism. Specifically, the results indicate that genes involved in carbon fixation, such as the rTCA cycle (*aclA*, *aclB*), CBB cycle (*rbcL*), 3HP bicycle (*prpE*), and DC/4HB pathway (*por*), are significantly influenced by acidification. As the acidity levels increase, the relative abundance of the predicted genes (*aclA*, *aclB*, *rbcL*, *prpE*, and *por*) also increases significantly (*P* < 0.05) based on PICRUSt2 predictions. Additionally, nitrogen metabolism pathways, including nitrogen fixation and denitrification, are significantly impacted by acidification. The abundance of *nifDKH* genes increases significantly with increasing acidity (*P* < 0.05), while genes involved in denitrification, such as *norBC* and *nosZ*, show the highest abundance in the control group, with a decrease in gene abundance as acidity levels rise (Fig. 4B, predicted based on PICRUSt2). In sulfur metabolism, apart from the dissimilatory sulfate reduction and oxidation pathways, other pathways demonstrate a decrease in gene abundance with increasing acidity levels (Fig. 4C, predicted based on PICRUSt2).

## DISCUSSION

### Ocean acidification affects the structure and function of the *Zostera japonica* rhizosphere bacterial community

Previous studies have shown that aquatic plants and large seaweeds exhibit a series of physiological responses when facing ocean acidification pressure, including changes in photosynthesis (55–57), respiration (58), reactive oxygen levels (59), and enzyme activities (60). In the preliminary experiments of this study, it was found that ocean acidification led to an increase in MDA content, indicating a transient oxidative damage to membrane systems, and the elevation of CAT activity indicated that *Zostera japonica* produced antioxidant substances capable of removing reactive oxygen species (36). Additionally, ocean acidification increased the concentration of photosynthetic pigments in seagrass leaves, while the photosynthetic rate exhibited a temporal pattern of initial decline followed by recovery. This trend likely reflects a response of seagrass to acidification stress.

External environmental factors, including those influenced by plant physiological changes, may further impact the structure and function of bacterial communities (7, 61, 62). This study found that ocean acidification significantly reduced the richness and diversity of the rhizosphere bacterial community of *Zostera japonica*. No significant difference in species richness was observed between the 1,000 ppm treatment and the control, whereas a clear trend emerged under higher levels of acidification. We speculate that, at 1,000 ppm, root exudates or the rhizosphere microenvironment may provide a buffering capacity that mitigates pH fluctuations, thereby temporarily alleviating the direct inhibitory effects of acidification on bacterial communities. As acidification intensifies, this buffering capacity is likely overwhelmed, leading to more pronounced impacts on bacterial community structure.

NMDS analysis and PERMANOVA results collectively confirmed that acidification treatments significantly altered the overall composition of the rhizosphere bacterial community, which is consistent with findings on the impact of ocean acidification on sediment microbial communities (63, 64). In terms of community composition, ocean acidification did not alter the dominant taxa in the rhizosphere bacterial community. However, the relative abundance of Bacteroidota systematically increased with increasing acidification levels, with *Tenacibaculum* significantly enriched (one-way ANOVA, *P* < 0.05). Bacteroidota typically contains genes encoding polysaccharide-degrading enzymes, giving them an advantage in degrading complex organic matter (65–67). *Tenacibaculum* are known to degrade various algal polysaccharides and chitin (68–70). The increase in its abundance may be related to changes in the organic carbon content of seagrass root exudates, such as mucilage and cell wall polysaccharides, under acidification conditions, suggesting that the rhizosphere environment may provide more specific organic carbon sources available for utilization, thus selecting for taxa with corresponding decomposition metabolic capabilities. Notably, the enrichment of Bacteroidota in the plant rhizosphere is not unique to seagrass ecosystems. In terrestrial plant rhizospheres, members of Bacteroidota possess conserved TonB-dependent transporter systems encoding xylan utilization loci, indicating that plant-derived hemicellulosic exudates (e.g., xylan) serve as important carbon sources (66). Additionally, the functional predictions of this study supported this hypothesis. Key genes (predicted based on PICRUSt2) in carbon fixation pathways, such as rTCA and CBB, showed a significant increase in abundance with increasing acidification levels (one-way ANOVA, *P* < 0.05), indicating enhanced assimilation potential of inorganic carbon by rhizosphere bacteria. We believe that acidification increases the concentration of dissolved inorganic carbon (DIC) in the rhizosphere environment, recruiting bacterial taxa with carbon fixation capabilities. Furthermore, the organic substances produced by the bacterial community through carbon fixation, along with potentially increased organic substrates secreted by seagrass roots, collectively provide resources for heterotrophic decomposer bacteria such as members of Bacteroidota, driving an increase in their abundance. It is worth noting that this study used PICRUSt2 to infer functional potential from 16S rRNA gene data. While widely applied, this approach cannot capture strain-specific functional variations, horizontal gene transfer, or actual gene expression levels. Future studies using metagenomics or metatranscriptomics are needed to validate the predicted functional shifts.

### Ocean acidification reshapes the interaction patterns and community assembly mechanisms of the rhizosphere bacterial community

The co-occurrence network results further revealed the impact of acidification on the internal interaction relationships within the bacterial community. Compared with the C and M groups, the H group exhibited reduced numbers of nodes and edges, along with lower modularity in the network. This pattern indicates that high-level acidification simplifies microbial interactions and reduces overall community complexity, consistent with the ecological hypothesis that environmental stress decreases microbial network complexity (71). Moreover, previous studies have generally suggested that higher modularity is associated with greater community stability (71–73). In this study, we likewise observed a decline in network modularity under the 2,000 ppm treatment, indicating reduced stability of the rhizosphere bacterial community under high acidification. Prior work has shown that decreases in species richness under environmental stress are a major driver of reduced network modularity (71). In highly stressful environments, communities tend to harbor fewer species and exhibit lower modularity, and these network characteristics collectively point to diminished community stability.

Of particular note, in the 1,000 ppm and 2,000 ppm treatment group, the number of species identified as network key taxa (module hubs and connectors) significantly increased, and these key taxa were mostly related to carbon and nitrogen cycling functions. This suggests that under extreme acidification conditions, the stability and function of the bacterial community may rely more on a few key taxa, which could maintain the overall community function through core metabolic functions, such as organic matter decomposition and nitrogen transformation. This corresponds to the importance of “core microbiomes” in plant stress resistance in ecology (74–77). This is also consistent with our functional prediction results, where the abundance of nitrogen fixation genes (*nifDKH*) significantly increased with acidification, while denitrification genes (*norBC*, *nosZ*) decreased. The differential response patterns of nitrogen metabolism functional genes, combined with the increase in key taxa related to nitrogen cycling in network analysis, indicate that under the selection of deterministic processes resulting in a new community structure, microbial taxa with nitrogen fixation functions occupy more critical network positions and functional niches.

Furthermore, the null model results demonstrated that under acidification conditions, deterministic processes dominated the community assembly of the *Zostera japonica* rhizosphere bacterial community. This suggests that ocean acidification, as an environmental filter, selectively recruits bacterial taxa within the *Zostera japonica* rhizosphere community. Additionally, as mentioned earlier, acidification-induced changes in host plant physiological responses (such as oxidative stress, photosynthetic adjustments, and secretions) indirectly altered the chemical microenvironment of the rhizosphere (such as O_2_ concentration and exudate composition), further exerting biologically related selective pressures. Environmental factor analysis also confirmed that acidification environment and host responses jointly shaped and influenced rhizosphere environmental characteristics, enhancing deterministic processes and selecting microbial taxa with specific functional genes (such as enhanced carbon fixation and nitrogen fixation genes, adaptive polysaccharide degradation genes), resulting in systematic changes in community composition, network structure, and assembly processes.

However, the experimental design in this study focused on the effects of ocean acidification, with temperature held constant across all treatments. Under high CO_2_ scenarios, increases in atmospheric CO_2_ are typically accompanied by significant warming of seawater. The absence of this co-varying factor limits our ability to directly extrapolate the present findings to future environmental conditions. Therefore, future experiments should incorporate factorial designs that combine multiple levels of acidification with temperature gradients, in order to disentangle the relative contributions and interactive effects of these two key global change drivers on seagrass-microbe interactions.

### Conclusion

This study analyzed the response characteristics of the *Zostera japonica* rhizosphere bacterial community to acidification from aspects such as community structure, function, and ecological processes. We found that acidification significantly reduced the richness and diversity of rhizosphere bacterial communities and altered their taxonomic composition. As acidification intensified, the influence of deterministic processes increased, exerting stronger selective pressures on microbial community assembly. Specifically, under high acidification conditions, the complexity and stability of bacterial co-occurrence networks declined. In addition, under 1,000 ppm and 2,000 ppm acidification treatments, the relative abundance of key taxa associated with carbon and nitrogen cycling increased. Furthermore, under high acidification conditions, the rhizosphere bacterial community showed enhanced assimilation capacity for inorganic carbon: the abundance of key genes associated with carbon fixation pathways (such as rTCA and CBB) significantly increased. At the same time, there was a shift toward enhanced nitrogen fixation and weakened denitrification in nitrogen metabolism functions, possibly reflecting microbial adjustments to the differences in carbon-to-nitrogen ratios under acidification. In summary, this study systematically revealed the multilayered response of the *Zostera japonica* rhizosphere bacterial community to ocean acidification from structure to function. These changes provide a theoretical basis for understanding the dynamics of seagrass-microbe interactions under climate change, as well as for predicting and managing seagrass bed ecosystems.

## Data Availability

The rhizosphere bacterial data set of this study has been deposited in the NCBI Sequence Read Archive under the accession number PRJNA1458631.

## SUPPLEMENTAL MATERIAL

Figure S1 aem.00277-26-s0001.tif Design diagram of the acidification experiment and the index of seawater and *Zostera japonica* leaves on the 40th day.

Supplemental legends aem.00277-26-s0002.docx Descriptive legends for all supplemental material.

Supplemental tables aem.00277-26-s0003.xlsx Tables S1 to S5.

ASM does not own the copyrights to Supplemental Material that may be linked to, or accessed through, an article. The authors have granted ASM a non-exclusive, world-wide license to publish the Supplemental Material files. Please contact the corresponding author directly for reuse.

## References

[1] Brodersen KE, Siboni N, Nielsen DA, Pernice M, Ralph PJ, Seymour J, Kühl M. 2018. Seagrass rhizosphere microenvironment alters plant-associated microbial community composition. Environ Microbiol 20:2854–2864. doi:10.1111/1462-2920.14245 29687545

[2] Ugarelli K, Chakrabarti S, Laas P, Stingl U. 2017. The seagrass holobiont and its microbiome. Microorganisms 5:81. doi:10.3390/microorganisms5040081 29244764 PMC5748590

[3] Xu L, Naylor D, Dong Z, Simmons T, Pierroz G, Hixson KK, Kim Y-M, Zink EM, Engbrecht KM, Wang Y, Gao C, DeGraaf S, Madera MA, Sievert JA, Hollingsworth J, Birdseye D, Scheller HV, Hutmacher R, Dahlberg J, Jansson C, Taylor JW, Lemaux PG, Coleman-Derr D. 2018. Drought delays development of the sorghum root microbiome and enriches for monoderm bacteria. Proc Natl Acad Sci USA 115:E4284–E4293. doi:10.1073/pnas.1717308115 29666229 PMC5939072

[4] Trivedi P, Leach JE, Tringe SG, Sa T, Singh BK. 2020. Plant-microbiome interactions: from community assembly to plant health. Nat Rev Microbiol 18:607–621. doi:10.1038/s41579-020-0412-1 32788714

[5] Zhang L, Zhang M, Huang S, Li L, Gao Q, Wang Y, Zhang S, Huang S, Yuan L, Wen Y, Liu K, Yu X, Li D, Zhang L, Xu X, Wei H, He P, Zhou W, Philippot L, Ai C. 2022. A highly conserved core bacterial microbiota with nitrogen-fixation capacity inhabits the xylem sap in maize plants. Nat Commun 13:3361. doi:10.1038/s41467-022-31113-w 35688828 PMC9187771

[6] Zeng Q, Hu H-W, Ge A-H, Xiong C, Zhai C-C, Duan G-L, Han L-L, Huang S-Y, Zhang L-M. 2025. Plant-microbiome interactions and their impacts on plant adaptation to climate change. J Integr Plant Biol 67:826–844. doi:10.1111/jipb.13863 39981843

[7] Li Y, Pan J, Zhang R, Wang J, Tian D, Niu S. 2022. Environmental factors, bacterial interactions and plant traits jointly regulate epiphytic bacterial community composition of two alpine grassland species. Science of The Total Environment 836:155665. doi:10.1016/j.scitotenv.2022.155665 35513157

[8] Wei N, Tan J. 2023. Environment and host genetics influence the biogeography of plant microbiome structure. Microb Ecol 86:2858–2868. doi:10.1007/s00248-023-02288-6 37610498

[9] Chen J, Sharifi R, Khan MSS, Islam F, Bhat JA, Kui L, Majeed A. 2022. Wheat microbiome: structure, dynamics, and role in improving performance under stress environments. Front Microbiol 12:821546. doi:10.3389/fmicb.2021.821546 35095825 PMC8793483

[10] Compant S, van der Heijden MGA, Sessitsch A. 2010. Climate change effects on beneficial plant-microorganism interactions. FEMS Microbiol Ecol 73:197–214. doi:10.1111/j.1574-6941.2010.00900.x 20528987

[11] Cavicchioli R, Ripple WJ, Timmis KN, Azam F, Bakken LR, Baylis M, Behrenfeld MJ, Boetius A, Boyd PW, Classen AT, et al.. 2019. Scientists’ warning to humanity: microorganisms and climate change. Nat Rev Microbiol 17:569–586. doi:10.1038/s41579-019-0222-5 31213707 PMC7136171

[12] French S, Levy-Booth D, Samarajeewa A, Shannon KE, Smith J, Trevors JT. 2009. Elevated temperatures and carbon dioxide concentrations: effects on selected microbial activities in temperate agricultural soils. World J Microbiol Biotechnol 25:1887–1900. doi:10.1007/s11274-009-0107-2

[13] Wahid F, Sharif M, Ali A, Fahad S, Adnan M, Noor M, et al.. 2020. Plant-microbes interactions and functions in changing climate, p 397–419. *In* Environment, climate, plant and vegetation growth. Springer.

[14] Parmesan C, Trisurat Y. 2022. Climate change 2022: Impacts, adaptation and vulnerability. GIEC.

[15] Barros V R, Broome J, Cramer W, Christ R, et al.. 2014. Climate change 2014: synthesis report. *In* Contribution of working groups I, II and III to the fifth assessment report of the intergovernmental panel on climate. Ipcc, Change.

[16] Arístegui J, Hallberg R. 2022. Changing ocean, marine ecosystems, and dependent communities

[17] Caldeira K, Wickett ME. 2003. Oceanography: anthropogenic carbon and ocean pH. Nature 425:365–365. doi:10.1038/425365a 14508477

[18] Kroeker KJ, Kordas RL, Crim RN, Singh GG. 2010. Meta‐analysis reveals negative yet variable effects of ocean acidification on marine organisms. Ecol Lett 13:1419–1434. doi:10.1111/j.1461-0248.2010.01518.x 20958904

[19] Stewart‐Sinclair PJ, Last KS, Payne BL, Wilding TA. 2020. A global assessment of the vulnerability of shellfish aquaculture to climate change and ocean acidification. Ecol Evol 10:3518–3534. doi:10.1002/ece3.6149 32274006 PMC7141013

[20] Koch M, Bowes G, Ross C, Zhang X-H. 2013. Climate change and ocean acidification effects on seagrasses and marine macroalgae. Glob Chang Biol 19:103–132. doi:10.1111/j.1365-2486.2012.02791.x 23504724

[21] Kroeker KJ, Kordas RL, Crim R, Hendriks IE, Ramajo L, Singh GS, Duarte CM, Gattuso J-P. 2013. Impacts of ocean acidification on marine organisms: quantifying sensitivities and interaction with warming. Glob Chang Biol 19:1884–1896. doi:10.1111/gcb.12179 23505245 PMC3664023

[22] Qin L-Z, Suonan Z, Kim SH, Lee K-S. 2021. Coastal sediment nutrient enrichment alters seagrass blue carbon sink capacity. Environ Sci Technol 55:15466–15475. doi:10.1021/acs.est.1c03782 34698488

[23] Borum J, Pedersen O, Kotula L, Fraser MW, Statton J, Colmer TD, Kendrick GA. 2016. Photosynthetic response to globally increasing CO _2_ of CO‐occurring temperate seagrass species. Plant Cell Environ 39:1240–1250. doi:10.1111/pce.12658 26476101

[24] Ow YX, Collier CJ, Uthicke S. 2015. Responses of three tropical seagrass species to CO2 enrichment. Mar Biol 162:1005–1017. doi:10.1007/s00227-015-2644-6

[25] Tsiola A, Krasakopoulou E, Daffonchio D, Frangoulis C, Tsagaraki TM, Fodelianakis S, Pitta P. 2023. Responses of free-living planktonic bacterial communities to experimental acidification and warming. Microorganisms 11:273. doi:10.3390/microorganisms11020273 36838238 PMC9963540

[26] Huang R, Zhang P, Zhang X, Chen S, Sun J, Jiang X, Zhang D, Li H, Yi X, Qu L, Wang T, Gao K, Hall-Spencer JM, Adams J, Gao G, Lin X. 2024. Ocean acidification alters microeukaryotic and bacterial food web interactions in a eutrophic subtropical mesocosm. Environ Res 257:119084. doi:10.1016/j.envres.2024.119084 38823617

[27] Huang Y-H, Lee C-L, Chung C-Y, Hsiao S-C, Lin H-J. 2015. Carbon budgets of multispecies seagrass beds at Dongsha Island in the South China Sea. Mar Environ Res 106:92–102. doi:10.1016/j.marenvres.2015.03.004 25797194

[28] Gillanders B M. 2006. Seagrasses, fish, and fisheries, p 503–505. *In* Seagrasses: Biology, Ecologyand Conservation. Springer.

[29] Tarquinio F, Hyndes GA, Laverock B, Koenders A, Säwström C. 2019. The seagrass holobiont: understanding seagrass-bacteria interactions and their role in seagrass ecosystem functioning. FEMS Microbiol Lett 366:fnz057. doi:10.1093/femsle/fnz057 30883643

[30] Vogel MA, Mason OU, Miller TE. 2021. Composition of seagrass phyllosphere microbial communities suggests rapid environmental regulation of community structure. FEMS Microbiol Ecol 97. doi:10.1093/femsec/fiab013 33493257

[31] Philippot L, Raaijmakers JM, Lemanceau P, van der Putten WH. 2013. Going back to the roots: the microbial ecology of the rhizosphere. Nat Rev Microbiol 11:789–799. doi:10.1038/nrmicro3109 24056930

[32] Bais HP, Weir TL, Perry LG, Gilroy S, Vivanco JM. 2006. The role of root exudates in rhizosphere interactions with plants and other organisms. Annu Rev Plant Biol 57:233–266. doi:10.1146/annurev.arplant.57.032905.105159 16669762

[33] Majeed A, Muhammad Z, Ahmad H. 2018. Plant growth promoting bacteria: role in soil improvement, abiotic and biotic stress management of crops. Plant Cell Rep 37:1599–1609. doi:10.1007/s00299-018-2341-2 30178214

[34] Moreau D, Bardgett RD, Finlay RD, Jones DL, Philippot L. 2019. A plant perspective on nitrogen cycling in the rhizosphere. Funct Ecol 33:540–552. doi:10.1111/1365-2435.13303

[35] Zhang J, Yang Q, Yue W, Yang B, Zhou W, Chen L, Huang X, Zhang W, Dong J, Ling J. 2023. Seagrass Thalassia hemprichii and associated bacteria co-response to the synergistic stress of ocean warming and ocean acidification. Environ Res 236:116658. doi:10.1016/j.envres.2023.116658 37454799

[36] Wang H, Zang Y, Xin J, Li X, Xue S, Liang S, Tang X, Chen J. 2024. Exploring the leaf regeneration cycles response of Zostera japonica to ocean acidification. Science of The Total Environment 954:176830. doi:10.1016/j.scitotenv.2024.176830 39389131

[37] Lundberg DS, Lebeis SL, Paredes SH, Yourstone S, Gehring J, Malfatti S, Tremblay J, Engelbrektson A, Kunin V, Del Rio TG, Edgar RC, Eickhorst T, Ley RE, Hugenholtz P, Tringe SG, Dangl JL. 2012. Defining the core Arabidopsis thaliana root microbiome. Nature 488:86–90. doi:10.1038/nature11237 22859206 PMC4074413

[38] Costa R, Götz M, Mrotzek N, Lottmann J, Berg G, Smalla K. 2006. Effects of site and plant species on rhizosphere community structure as revealed by molecular analysis of microbial guilds. FEMS Microbiol Ecol 56:236–249. doi:10.1111/j.1574-6941.2005.00026.x 16629753

[39] Zhang X, Zhao C, Yu S, Jiang Z, Liu S, Wu Y, Huang X. 2020. Rhizosphere microbial community structure is selected by habitat but not plant species in two tropical seagrass beds. Front Microbiol 11:161. doi:10.3389/fmicb.2020.00161 32194512 PMC7065525

[40] Chen S, Zhou Y, Chen Y, Gu J. 2018. Fastp: an ultra-fast all-in-one FASTQ preprocessor. Bioinformatics 34:i884–i890. doi:10.1093/bioinformatics/bty560 30423086 PMC6129281

[41] Magoč T, Salzberg SL. 2011. FLASH: fast length adjustment of short reads to improve genome assemblies. Bioinformatics 27:2957–2963. doi:10.1093/bioinformatics/btr507 21903629 PMC3198573

[42] Barberán A, Bates ST, Casamayor EO, Fierer N. 2012. Using network analysis to explore co-occurrence patterns in soil microbial communities. ISME J 6:343–351. doi:10.1038/ismej.2011.119 21900968 PMC3260507

[43] Li M, Shao D, Zhou J, Gu J, Qin J, Chen W, Wei W. 2020. Signatures within esophageal microbiota with progression of esophageal squamous cell carcinoma. Chin J Cancer Res 32:755–767. doi:10.21147/j.issn.1000-9604.2020.06.09 33446998 PMC7797230

[44] Sun H, Wang T, Liu S, Tang X, Sun J, Liu X, Zhao Y, Shen P, Zhang Y. 2024. Novel insights into the rhizosphere and seawater microbiome of Zostera marina in diverse mariculture zones. Microbiome 12:27. doi:10.1186/s40168-024-01759-3 38350953 PMC10865565

[45] Segata N, Izard J, Waldron L, Gevers D, Miropolsky L, Garrett WS, Huttenhower C. 2011. Metagenomic biomarker discovery and explanation. Genome Biol 12:R60. doi:10.1186/gb-2011-12-6-r60 21702898 PMC3218848

[46] Douglas GM, Maffei VJ, Zaneveld JR, Yurgel SN, Brown JR, Taylor CM, Huttenhower C, Langille MGI. 2020. PICRUSt2 for prediction of metagenome functions. Nat Biotechnol 38:685–688. doi:10.1038/s41587-020-0548-6 32483366 PMC7365738

[47] Revelle W. 2015. Package “psych”. The comprehensive R archive network.

[48] Revelle W R. 2017. Psych: procedures for personality and psychological research

[49] Ali Abd Al-Hameed K. 2022. Spearman’s correlation coefficient in statistical analysis. International Journal of Nonlinear Analysis and Applications 13:3249–3255. doi:10.22075/ijnaa.2022.6079

[50] Liu Y, Gong L, Mu X, Zhang Z, Zhou T, Zhang S. 2020. Characterization and co-occurrence of microbial community in epiphytic biofilms and surface sediments of wetlands with submersed macrophytes. Science of The Total Environment 715:136950. doi:10.1016/j.scitotenv.2020.136950 32007899

[51] Ning D, Deng Y, Tiedje JM, Zhou J. 2019. A general framework for quantitatively assessing ecological stochasticity. Proc Natl Acad Sci USA 116:16892–16898. doi:10.1073/pnas.1904623116 31391302 PMC6708315

[52] Burns AR, Stephens WZ, Stagaman K, Wong S, Rawls JF, Guillemin K, Bohannan BJ. 2016. Contribution of neutral processes to the assembly of gut microbial communities in the zebrafish over host development. ISME J 10:655–664. doi:10.1038/ismej.2015.142 26296066 PMC4817674

[53] Fine PVA, Kembel SW. 2011. Phylogenetic community structure and phylogenetic turnover across space and edaphic gradients in western Amazonian tree communities. Ecography 34:552–565. doi:10.1111/j.1600-0587.2010.06548.x

[54] Oksanen J, Kindt R, Legendre P. 2013 Package ‘vegan’. Community ecology package 2:1–295.

[55] Ashraf M, Harris PJC. 2013. Photosynthesis under stressful environments: an overview. Photosynt 51:163–190. doi:10.1007/s11099-013-0021-6

[56] Hussner A, Mettler‐Altmann T, Sand‐Jensen K. 2016. Acclimation of photosynthesis to supersaturated CO 2 in aquatic plant bicarbonate users. Freshw Biol 61:1720–1732. doi:10.1111/fwb.12812

[57] Gao G, Beardall J, Bao M, Wang C, Ren W, Xu J. 2018. Ocean acidification and nutrient limitation synergistically reduce growth and photosynthetic performances of a green tide alga Ulva linza. Biogeosciences 15:3409–3420. doi:10.5194/bg-15-3409-2018

[58] Millar AH, Whelan J, Soole KL, Day DA. 2011. Organization and regulation of mitochondrial respiration in plants. Annu Rev Plant Biol 62:79–104. doi:10.1146/annurev-arplant-042110-103857 21332361

[59] Xu H, Pang T, Zhang L, Liu J. 2025. Photosynthetic performance of the red algae Gracilariopsis lemaneiformis under high seawater pH: excess reactive oxygen production due to carbon limitation. Photochem Photobiol 101:239–250. doi:10.1111/php.13968 39032084

[60] Fernández PA, Roleda MY, Hurd CL. 2015. Effects of ocean acidification on the photosynthetic performance, carbonic anhydrase activity and growth of the giant kelp Macrocystis pyrifera*.* Photosynth Res 124:293–304. doi:10.1007/s11120-015-0138-5 25869634

[61] Wu Z, Liu Q, Li Z, Cheng W, Sun J, Guo Z, Li Y, Zhou J, Meng D, Li H, Lei P, Yin H. 2018. Environmental factors shaping the diversity of bacterial communities that promote rice production. BMC Microbiol 18:51. doi:10.1186/s12866-018-1174-z 29866052 PMC5987589

[62] Berg G, Smalla K. 2009. Plant species and soil type cooperatively shape the structure and function of microbial communities in the rhizosphere. FEMS Microbiol Ecol 68:1–13. doi:10.1111/j.1574-6941.2009.00654.x 19243436

[63] Su X, Yang X, Li H, Wang H, Wang Y, Xu J, Ding K, Zhu Y. 2021. Bacterial communities are more sensitive to ocean acidification than fungal communities in estuarine sediments. FEMS Microbiol Ecol 97. doi:10.1093/femsec/fiab058 33792671

[64] Currie AR, Tait K, Parry H, de Francisco-Mora B, Hicks N, Osborn AM, Widdicombe S, Stahl H. 2017. Marine microbial gene abundance and community composition in response to ocean acidification and elevated temperature in two contrasting coastal marine sediments. Front Microbiol 8:1599. doi:10.3389/fmicb.2017.01599 28878754 PMC5572232

[65] Chen ZT, Yu B, Li YS, Zhu ZW, Zhang XY, Li Y. 2025. Christiangramia qingdaonensis sp. nov., a novel polysaccharide-degrading Bacteroidota bacterium, isolated from intertidal sediment. In Review. doi:10.21203/rs.3.rs-8043186/v1 41495283

[66] Martin H, Rogers LA, Moushtaq L, Brindley AA, Forbes P, Quinton AR, Murphy ARJ, Hipperson H, Daniell TJ, Ndeh D, Amsbury S, Hitchcock A, Lidbury IDEA. 2025. Metabolism of hemicelluloses by root-associated bacteroidota species. ISME J 19:wraf022. doi:10.1093/ismejo/wraf022 39913342 PMC11892949

[67] Liu T-H, Zhou H-Y, Zhang H-Z, Wang F-Q, Liu Y-Z, Teng J-H, Fan S-J, Lu D-C, Du Z-J. 2025 Genomic insights into polysaccharide substrate utilization and novel genera resource mining in macroalgal epiphytic bacteria. bioRxiv. doi:10.1101/2025.04.07.647558

[68] Kristyanto S, Kim KR, Jung J, Kim HM, Kim K, Jeon CO. 2022. Tenacibaculum aquimarinum sp. nov., isolated from a marine alga and seawater. Int J Syst Evol Microbiol 72:005477. doi:10.1099/ijsem.0.005477 35943445

[69] Oh YS, Kahng H Y, Lee D H. 2012. Parasphingopyxis marina sp. nov. isolated from coastal seawater. Int J Syst Evol Microbiol 62:414–419. doi:10.1099/ijs.0.030114-0 21460140

[70] Xu Z-X, Yu P, Mu D-S, Liu Y, Du Z-J. 2017. Tenacibaculum agarivorans sp. nov., an agar-degrading bacterium isolated from marine alga Porphyra yezoensis Ueda. Int J Syst Evol Microbiol 67:5139–5143. doi:10.1099/ijsem.0.002432 29043952

[71] Hernandez DJ, David AS, Menges ES, Searcy CA, Afkhami ME. 2021. Environmental stress destabilizes microbial networks. ISME J 15:1722–1734. doi:10.1038/s41396-020-00882-x 33452480 PMC8163744

[72] de Vries FT, Griffiths RI, Bailey M, Craig H, Girlanda M, Gweon HS, Hallin S, Kaisermann A, Keith AM, Kretzschmar M, Lemanceau P, Lumini E, Mason KE, Oliver A, Ostle N, Prosser JI, Thion C, Thomson B, Bardgett RD. 2018. Soil bacterial networks are less stable under drought than fungal networks. Nat Commun 9:3033. doi:10.1038/s41467-018-05516-7 30072764 PMC6072794

[73] Luo S, Png GK, Ostle NJ, Zhou H, Hou X, Luo C, Quinton JN, Schaffner U, Sweeney C, Wang D, Wu J, Wu Y, Bardgett RD. 2023. Grassland degradation-induced declines in soil fungal complexity reduce fungal community stability and ecosystem multifunctionality. Soil Biology and Biochemistry 176:108865. doi:10.1016/j.soilbio.2022.108865

[74] Liu S, Wu J, Cheng Z, Wang H, Jin Z, Zhang X, Zhang D, Xie J. 2025. Microbe-mediated stress resistance in plants: the roles played by core and stress-specific microbiota. Microbiome 13:111. doi:10.1186/s40168-025-02103-z 40320520 PMC12051278

[75] Du S, Li X-Q, Feng J, Huang Q, Liu Y-R. 2023. Soil core microbiota drive community resistance to mercury stress and maintain functional stability. Science of The Total Environment 894:165056. doi:10.1016/j.scitotenv.2023.165056 37348729

[76] Toju H, Peay KG, Yamamichi M, Narisawa K, Hiruma K, Naito K, Fukuda S, Ushio M, Nakaoka S, Onoda Y, Yoshida K, Schlaeppi K, Bai Y, Sugiura R, Ichihashi Y, Minamisawa K, Kiers ET. 2018. Core microbiomes for sustainable agroecosystems. Nat Plants 4:247–257. doi:10.1038/s41477-018-0139-4 29725101

[77] Aqueel R, Badar A, Roy N, Ijaz UZ, Malik KA. 2024. Disease resistance correlates with core microbiome diversity in cotton. Curr Microbiol 81:302. doi:10.1007/s00284-024-03827-1 39115581 PMC11310248

