# Supplementary figures and images for: Response of Zostera japonica rhizosphere bacteria to ocean acidification
Source: Appl Environ Microbiol. 2026 Jun 29;92(7):e00277-26. doi: 10.1128/aem.00277-26 (PMC13390396; doi:10.1128/aem.00277-26)

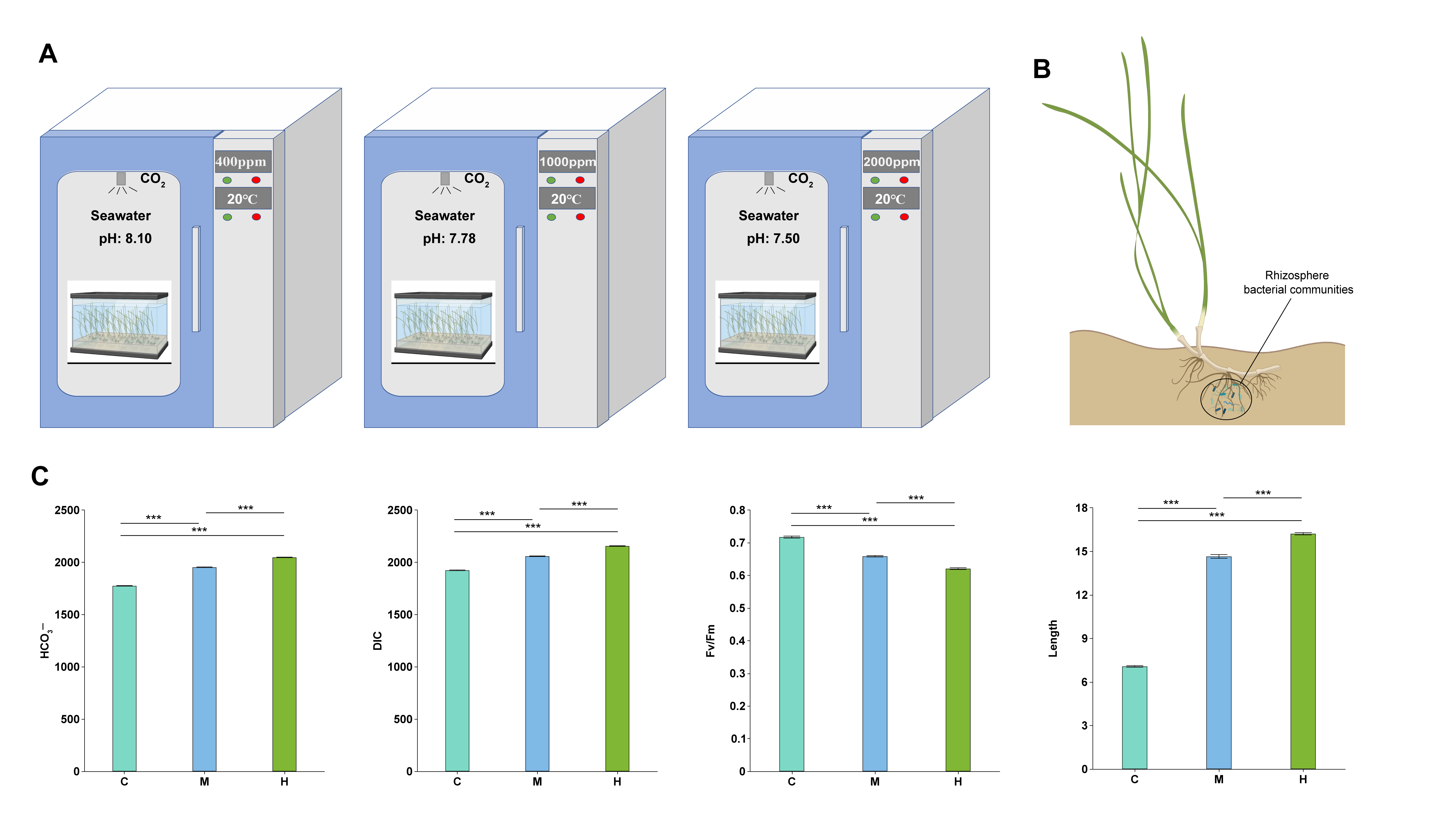

Supplement: Figure S1 — Design diagram of the acidification experiment and the index of seawater and Zostera japonica leaves on the 40th day. [file aem.00277-26-s0001.tif]
