# Supplementary material for: Response of Zostera japonica rhizosphere bacteria to ocean acidification
Source: Appl Environ Microbiol. 2026 Jun 29;92(7):e00277-26. doi: 10.1128/aem.00277-26 (PMC13390396; doi:10.1128/aem.00277-26)
Supplement: Supplemental legends — Descriptive legends for all supplemental material. [file aem.00277-26-s0002.docx]

**Supplemental Material Legends**

**Figure S1.** The design diagram of acidification experiment and the index of seawater and *Zostera japonica* leaves on the 40^th^ day. (A) Schematic diagram of the acidification experimental system. (B) Sampling zones for rhizosphere bacterial communities of *Zostera japonica*. (C) Environmental factors (seawater parameters) and physiological indices of *Zostera japonica* leaves under different acidification treatments.

**Supplementary Table 1.** Bacterial community sequencing data of all of the samples.

**Supplementary Table 2.** Results of *Zostera japonica* bacterial community compositions predicted by PERMANOVA models.

**Supplementary Table 3.** The properties of co-occurrence networks of bacterial communities under different acidification treatments.

**Supplementary Table 4.** List of keystone nodes and their taxonomic information under different acidification treatments.

**Supplementary Table 5.** Measured seawater physicochemical parameters and physiological indices of *Zostera japonica* under three acidification treatment conditions.
